# Digital monitoring for data-driven antimicrobial stewardship: a process perspective from resource-constrained contexts in India

**DOI:** 10.3389/frabi.2023.1214826

**Published:** 2023-08-02

**Authors:** Yogita Thakral

**Affiliations:** ^1^ The Health Information Systems Programme (HISP) Centre and Department of Informatics, University of Oslo, Oslo, Norway; ^2^ Society of Health Information Systems Programme, Health Information Systems Programme (HISP) India, New Delhi, India

**Keywords:** antimicrobial resistance, antimicrobial stewardship, digital intervention, information system, resource constrained, microbiology, AST

## Abstract

Antimicrobial Resistance (AMR) is one of society’s most urgent global issues, requiring urgent multidisciplinary-based research and practice approaches to engage with these policies. Several global and national policy statements have been released in the last two decades, particularly emphasising the strengthening of the digital surveillance system. However, implementing these initiatives remains patchy, particularly in the context of public health systems in Low- and Middle-Income Countries. This paper argues that one of the significant reasons contributing to this sub-optimal uptake of these systems is that the top-down implementation models do not adequately cater to the needs, aspirations, and capacities of the health facility staff, who, ultimately, are the end users of the system. The paper highlights the importance of digital technology in healthcare facilities with resource constraints to promote the responsible use of antibiotics. It discusses the process of developing an evidence base for action in low- and middle-income countries (LMICs) through digitally mediated data-driven policy. This process is conceptualised as a three-phase process, which involves stabilising data entry, generating outcomes, and taking action at the local level. The paper argues the need for bottom-up implementation models, which emphasise the need to understand the practices users engage with in their everyday work and design the digital system to add value and not work to these everyday practices. The paper emphasises the importance of building local capacities to develop effective and sustainable antimicrobial stewardship (AMS) programs through enabling networking around digital solutions, creating value in networked partnerships, initiating conversations around data, and raising awareness of the digital to develop AMS programs.

## 1 Introduction

Antimicrobial Resistance (AMR) is one of the most significant health challenges facing the world today, particularly in low- and middle-income countries (LMICs) already grappling with existing inequities in access to and quality of care across various socioeconomic, geographic, and demographic categories (WHO, 2022). The causes and effects of AMR are interconnected and mutually reinforcing, with AMR being both a cause and a consequence of existing inequities in LMICs. In India, for example, high disease burden, limited resources, and weak health systems result in the inappropriate use of antibiotics, which is the leading driver of AMR, thereby exacerbating the existing inequities (Charani et al., 2021). Several policies and frameworks are defined at the global and national levels. For instance, the Global action plan (GAP) developed by WHO guided the development of National action plans (NAPs) in partner countries. It discusses the key strategies to address AMR at national and global levels. All NAPs emphasise the importance of using antimicrobials^1^ carefully and appropriately (Charani et al., 2023). However, they do not take into account the contextual challenges and obstacles hindering progress towards this goal, especially in resource-constrained settings already grappling with challenges (Iskandar et al., 2021). These challenges include inadequate legal and regulatory frameworks, lack of electronic health record systems, difficulties with access to quality-assured medicines, and, over-the-counter (OTC) availability of antibiotics,. The availability of regular data monitoring and analysis is of utmost importance in addressing Antimicrobial Resistance (AMR). By systematically collecting and analysing data on antimicrobial use, resistance patterns, and associated factors, healthcare systems and policymakers gain critical insights into the magnitude and trends of AMR within their regions. Regular data monitoring allows for the identification of emerging resistance patterns, high-risk areas, and specific populations that may be disproportionately affected. However, the efforts to combat AMR are challenged by the poor availability of reliable data (Van Boeckel et al., 2014; O’Neill, 2016; Murray et al., 2022), particularly from low and middle-income countries (Laxminarayan, 2022), making it very difficult to estimate the burden of disease (de Kraker et al., 2016).

Initiating antibiotics policy-making in LMICs is a complex process that requires navigating through various challenges, particularly in the area of AMR surveillance. Addressing these challenges requires building laboratory capacity, strengthening surveillance systems, and developing a comprehensive framework for managing antimicrobial resistance. The availability of information is a critical component to identifying the contextual challenges to address the challenge of over and irrational use of antibiotics. One of the key strategic priorities in NAPs is to *strengthen the knowledge and evidence base through digital surveillance and monitoring to strengthen policy and practice.* Strengthening AMR surveillance is critical for ensuring evidence-based policy-making and supporting effective strategies to promote the responsible use of antibiotics for controlling the spread of antimicrobial resistance. However, owing to the poor digital infrastructure, most LMICs lack the capability to capture routine health information to understand the existing health disparities effectively (Marmot, 2015). For example, in India, the surveillance of AMR is fragmented, with three independent networks^2^ collecting surveillance data based on antimicrobial susceptibility test (AST) results mainly from tertiary hospitals, which is not representative of the resistance profile of the entire country. It lacks uniformity and is not scalable geographically and functionally, and faces challenges of uniformity in data, incomplete information with essential fields like the demographic details and crucial antibiotics missing (Sonal et al., 2023). Addressing these challenges requires a profound contextual understanding of the everyday practices of the staff in the health institution, and how novel digital capabilities can be introduced to support AMR surveillance for evidence-based policy making. For instance, the everyday challenges faced at the microbiology labs of the hospitals in capturing and analysing the information, the challenges in digital management of data and the challenges at the hospitals to use the information for action for patient care and policy making.

The availability of information about how and why antibiotics are being used could inform efforts to drive improvements. This contextual understanding can guide the process of development of information systems, shaping new practices and processes that guide patient care and policy-making at local levels with the ability to be scalable to other contexts (Sahay et al., 2020). Antimicrobial stewardship (AMS) programs aim to improve the quality and safety of antimicrobial use, but the effective implementation of these programs requires a two-pronged approach. Firstly, a sociocultural approach is needed to understand the contextual factors that affect the capturing, analysing and using information at the facility level. Secondly, a technical approach to understand the health information challenges that can guide the design and implementation of digital monitoring systems to make the information available about the contextual challenges of antibiotics use, resistance patterns, dosing, duration of treatment etc. Combining both the sociocultural and technical approaches is crucial for the success of AMS programs in promoting the appropriate use of antimicrobials and reducing the development of antimicrobial resistance.

Drawing from a sociocultural and technical approach, this paper describes and addresses these two facets of the challenge of AMR data management by discussing; *i) what are the novel data management practices to strengthen AMR surveillance at a facility level, and ii) how can these practices be translated to initiate the development of a data-driven policy for AMS?* In answering these questions, this paper describes the long path towards the development of AMS programs in resource constrained contexts. This paper develops a granular view of practices and examines them under three interconnected phases of the process to initiate the development of data-driven policy, i) practices to make the “invisible” more visible? ii) practices to enhance the circulation of this visibility across the health institution, and iii) practices to act on this information for changing policy and practice, within and across hospitals.

## 2 Materials and methods

This is an ongoing qualitative longitudinal study started in 2019 in a tertiary hospital in a north Indian state. The hospital under study, typical of most public hospitals, has limited experience with digital systems and suffers from constraints of weak diagnostics, limited capacity, manpower, and infrastructure, with information on antibiotics prescriptions and consumption currently largely invisible. The hospital has a high patient load (of around 2000 patients every day) that while struggling with historically existing constraints of manpower, resources, and digital infrastructure, took the decision to implement a digital ABR reporting system.

The process started with two parallel studies, i) a study of the challenges associated with AMR data management at the hospital, ii) the design and development of an AMR surveillance system at the microbiology lab of the hospital based on a free and open-source digital platform called DHIS2 (see dhis2.org) with technical support of a local NGO called HISP (Health Information Systems Programme) India. This study uses a practice lens (Orlikowski, 2010) to understand the everyday work associated with AMR data management, which was identified, documented, and discussed, which provided insights into the system design.

The author of this paper holds dual roles as a researcher and a member of the HISP team. As a researcher, the author studied the practices of antibiotics use and exploring how digital systems can effectively intervene in this process. At HISP India, the author is actively involved in the practical processes of designing, developing, and implementing the digital antimicrobial resistance (ABR) monitoring system. The author’s unique position allows her to observe the real-world practices of antibiotic use in context while also analysing them through the lens of research. This enables bringing a critical perspective to the development and implementation of the ABR monitoring system and to identify areas where improvements can be made.

The data collection to study the challenges associated with AMR data management and the design and implementation of an AMR monitoring system were parallel activities and hence discussed together.

### 2.1 Data collection

Data collection and the design and development of the digital monitoring application are carried out as ongoing activities. To understand the challenges associated with AMR data management and ongoing design and development of AMR surveillance system, I studied the overall information flow at the hospital about how AMR data is generated, where it is generated, and how it reaches the treating physician and the patient, and the practices of the stakeholders involved in AMR data handling. The data collection included interviews and discussions with the hospital stakeholders, with a key focus on the microbiology lab, to understand the practices around sample testing, starting from the arrival of the patient at the hospital, the physician ordering for the AST, sample collection, sample transfer, testing at the lab, documentation, dissemination, and data use. This empirical work fed into the design and implementation of the AMR surveillance system. **Table 1** provides an overview of the data collection tools of AMR data management, and in the next section, I will discuss data collection in detail:

**Table 1:** Data collection tools.

Stakeholder group	Data collection methods	Numbers	Details
Staff at the registration and billing counter	Interviews	2	Workflow and activities involved during registration, billing, sending the patient for consultation, collecting the sample and getting test results from the lab. Challenges faced with the existing workflow.
Staff at the sample collection unit responsible for collection and transfer		2	Existing methods of sample collection, transfer to various labs, challenges faced
Physicians		10	Information needed for patient care, if it is readily available, what information is needed, preferable format for reporting, challenges with the existing workflow, suggestions for improvements
Microbiologists		5	Data reporting and details of reporting to physicians and the state/reports dissemination to patients/physicians.
Lab technicians		3	Receiving samples, maintaining records, testing, etc., at the lab.
Data entry operator (DEO) at the lab		2	Data entry and flow of information using the application and challenges in cases the test is delayed, missing information in registers, etc.
Registration staff, sample collection staff, Microbiology team, Lab technicians, DEO	Observation	AMR data handling in the registers and digital application; Billing and sample collection; Microbiology lab-data entry, reports dissemination, data use	
Physicians, microbiologists, staff at the microbiology department, and the Principal of the hospital	Discussion	During the design, development, implementation, and efforts to use the data.	
Microbiology teams, Physicians, Hospital management, HISP India (technical) team, Microbiology team from other hospitals in the state	Workshop	Annual workshop for requirements analysis, implementation progress, challenges and the way forward.	

#### 2.1.1 Semi-structured interviews

The interviews were important sources of empirical material. In some of these interviews, the interaction between the researcher and informant was a planned event and was structured as an interview. Often, however, the data collection was more *ad-hoc*, which resulted because of observation or a discussion. The microbiologists, lab technicians, and data entry operators were first interviewed using a semi-structured interview guide to understand their respective workflow at the billing and registration department, sample collection unit at the hospital, and microbiology lab. Subsequent interviews were scheduled either because of observations or follow-up interviews from discussions. Open discussions were held with the physicians during the designing of the reports for their feedback on the design during the development and also after the development.

#### 2.1.2 Observation of AMR data management practices

**Figure 1:**
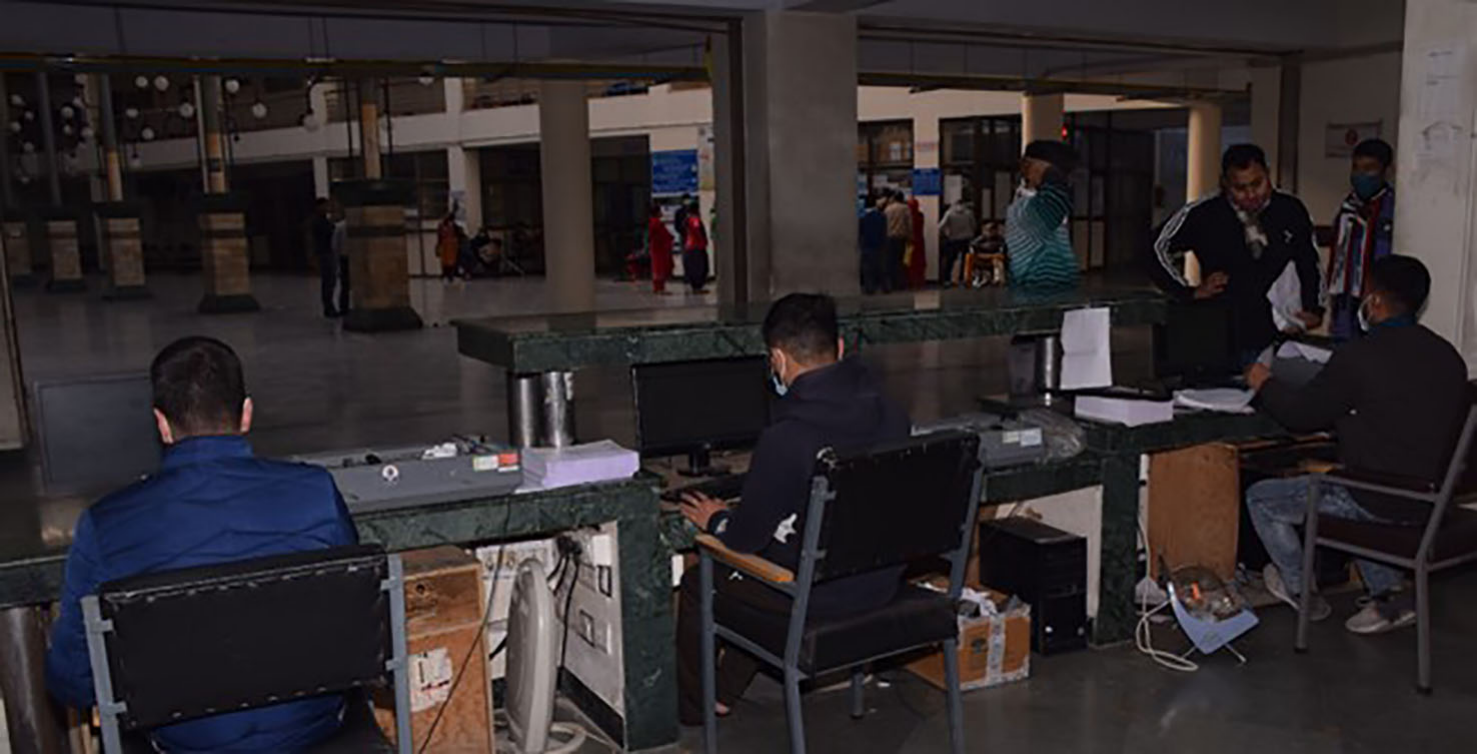
Registration and billing counter at the hospital.

Along with the interviews, observing the workflow starting from the patient’s arrival at the registration desk at the hospital (**Figure 1**) was an important source of data collection. The observations were limited to understanding the AMR data handling practices - how patient data was captured and reached the physician through the microbiology lab during treatment at the hospital. The purpose of being physically present in the hospital, rather than relying solely on interviews, was to witness their organization and practices, contributing to an in-depth contextual understanding of the flow of information. These dynamics have been important in shaping knowledge from interviews (Mason, 2017).

#### 2.1.3 Annual workshop

An annual workshop was held every year at the hospital premises organized by both the hospital and the HISP India team to discuss the progress implementation challenges, and way forward. It was an important data collection tool to both observe and discuss the progress of the implementation over a period of years and to hear the views of both the microbiology team and the hospital management.

The annual workshop was especially relevant, as the diversity and number of participants over the years increased as the microbiology teams from the nearby hospitals and physicians from several departments in the hospital joined. This helped in gaining a deep insight into the relevance and also the change in the ownership of the application and the data at the hospital by the microbiology team and the hospital and a general sense of AMR data from other stakeholders.

#### 2.1.4 Training sessions

Training sessions, both onsite and online, were organized every few months as the application design and implementation followed a quick prototyping approach to provide the microbiology team with the essential features they needed. These training sessions were both important for rapport building initially and later to understanding their requirements and observing their changing views of the digital monitoring application as their desired features were incorporated over a period of time. Training sessions were also an important tool to build the capacity of the microbiology team and observe their requirements becoming more refined as they gained experience using digital technology for data capture and report generation over time.

#### 2.1.5 Weekly meetings

Weekly meetings were held mostly online and sometimes on-site, depending on the field visits. The weekly meetings were an essential tool for understanding the challenges the microbiology team and the data entry operator were facing while capturing the data and generating reports. The microbiology team shared their requirements for features and challenges with using the features and data from the application. Technical, operational, and other challenges were shared, and the HISP team presented new features and sequentially implemented them. These meetings were an important tool for assessing the progress at short intervals and building the local capacity of the microbiology team to test the application when new features were added and to generate their own reports regularly. As the application started taking stronger roots and the data entry became increasingly stable, discussions with physicians and hospital staff on aspects of the use of the reports were initiated.

### 2.2 Data analysis

The data analysis followed sequential steps:

***Data collation and organization*:** All data collected including interview notes, observations, and study of documents were organized and collated to facilitate analysis. ***Transcriptions***: All primary data was transcribed and translated from Hindi to English wherever needed, and digitized. ***Thematic analysis to identify the AMR data management challenges***: First, responses were grouped, and major practices were identified representing the understanding of the problem. For example, a major theme identified from the analysis of physicians’ interviews was the lack of information to decide the antibiotic treatment. The unavailability of information for patient treatment and for preparing local guidelines in the form of antibiotics policy was addressed through a digital reporting system that potentially would make available testing profiles to physicians to guide their prescription decisions. The base for generating these testing profiles was the automation of the testing process (called AST – Antibiotic Susceptibility Tests) which was carried out in the microbiology lab. The process included the *initial requirements construction, followed by continuous ongoing and intertwined processes of design improvements, implementation, and regular evaluations.*

## 3 Results

In this section, first the results from the study of challenges associated with AMR data management are described. This is followed by a description of the practices identified subsequently leading to the development of data-driven policy, i) practices to make the “invisible” more visible, ii) practices to enhance the circulation of this visibility across the health institution, and iii) practices to act on this information for changing policy and practice, within and across hospitals.

### 3.1 AMR data management practices

The hospital under study had limited prior experience with digital systems. The process of recording patients’ data, testing samples, maintaining records, and sharing reports with patients and physicians was manual and time-consuming. A microbiologist mentioned:

*Once the test results are ready, they are documented on paper and in the register. Patients or their attendants come to the lab to collect the results, and the rest of the test results are kept on a table once the lab closes every day at 15:00 for patients to come and collect themselves.*

To carry out the AST test, the microbiologists mentioned that they sometimes lacked the stock of antimicrobial discs to test the samples received at the lab. In the absence of antimicrobial discs, certain antimicrobials could not be tested for some bacteria that were crucial to be tested for certain infections. There were regular stock delays and operational challenges, which prevented microbiologists from generating quality data. One microbiologist explained:

*We have asked the central store for a stock of cefixime from the last two months, but we have not received the stock yet. In situations when we do not have an antibiotic plate for testing, we usually test other antibiotics from the panel because it anyway takes multiple stock requests and a very long time to get the stock.*

This challenge of microbiologists to perform the tests for certain antibiotics also affected physicians’ prescription practices and supplemented their reasons not to prescribe an AST test. A physician described:

*A lot of patients come with a complaint of obstructive pulmonary disease, and in such cases, I need the resistance pattern for cefixime to see if I should prescribe it or should I prescribe a higher-generation antibiotic. But I do not see the resistance pattern for cefixime in the results. The test results are received late and incomplete. So, I prescribe based on my own experience.*

The challenge of delayed test results amplifies by the incomplete information the microbiologists were provided with. The lab technicians at the microbiology lab complained about the quality of the information received in the requisition forms with samples to be tested. These forms were frequently incomplete, with missing patients and sample details. The form had a code of the sample that was mentioned on the vial containing the sample and the name of the patient. However, other details like the department where the patient is treated, his/her diagnosis, and many other related fields were often blank. These issues added to the delays in getting test results. The microbiology lab found it difficult to document and sometimes even test such samples with the basic information being unavailable. A lab technician exclaimed:

*It is really difficult to test because of incomplete information. Sometimes the sample type and the patient’s CR number (unique number) are missing from the form. It is not possible to start the test without these basic details, and the sample is sent back to the collection unit for more information.*

The hospital did not have existing guidelines for antibiotic use or infection control to guide physicians of how to prepare treatment plans. During the discussions, the head of the microbiology department said:

*We have all the data lying in registers, and these registers are not digitized. Whenever someone asks for some information on a particular pathogen, we look at the registers and collect information from them, and share. To develop an infection control or an antibiotic policy, a huge amount of analysed data from all pathogens is needed, which is not available right now.*

The microbiology team was initially unable to generate an aggregate report to be shared with the hospital management and state authorities due to the manual and fragmented nature of data records. A microbiologist at the hospital explained:

*It is impossible to generate a report with the data in the registers. We can count estimates based on the data for specific pathogens, but a holistic analysis of the test results is impossible to generate with manual analysis. There are three registers each for Blood, Urine, and all other samples, and to prepare an aggregated report, the data from all registers need to compile separately and then aggregated.*

Another microbiologist at the lab mentioned:

*Finding relevant data manually from all the registers and then aggregating them together from all registers is a tedious exercise. The team does not have the time to do this task, and there is already a manpower crunch at the lab. We only have two lab technicians who do all the work, and they need to stay overtime to start the culture for the samples received later during the day. Data analysis becomes secondary when there is a huge daily task that needs to be done.*

Limited capacity and resources added to the existing challenges of delayed test results. Studies of practices of antibiotics use and AMR data handling guided the identification of the class of problems.

### 3.2 Practices to make the “invisible” more visible

In this case, the term invisible refers to the unavailability of actionable data at both the practice and policy levels for local action at the hospital. The unavailability of data is made worse by the constraints of capacity, resources, and lack of experience with the digital systems accompanied by less awareness about the problem of AMR in the public health system.

At the micro level of the hospital, the practice of managing the AMR data at the microbiology lab is largely manual. The lab has manual registers where the technicians record the patients’, samples, and test results daily before being relayed back to the indenting department. Existing data in the manual registers was not subjected to any systematic analysis before the introduction of the digital application and was now captured and analyzed to identify the resistance patterns in the hospital. However, this journey of transformation involved work and transformation of the existing practices of manual data collection and management to enable the digital system to make the problem of AMR visible.

The initial focus was mainly on i) recording the AST requisition form sent by hospital physicians to the lab, and ii) recording the test results. Subsequently, other modules have been incrementally added (for example, data analysis, and infection control) based on continuous discussion and interactions with the team in the lab. Several challenges were observed after the introduction of the digital system that required changes in the existing practices to stabilize the use of the digital application. An important initial challenge was to strengthen the practices by which the missing details in the requisition form, such as the patient’s clinical symptoms, diagnosis, antibiotics treatment, and sometimes even the sample type and location, could be filled. A microbiologist told during an interview:

The requisition form received with each sample from either the sample collection unit for outpatients or wards/in-patient departments is incomplete in many cases or is illegible. There is no information on the diagnosis, proposed antibiotic treatment plan, etc. advised before ordering AST.

This missing information needed to be filled in to generate more meaningful reports, such as resistance patterns by departments and sending test results to the treating physicians and ultimately the patients. The software team worked closely with the microbiology team to identify missing fields by creating a report, which was then presented to the microbiologists.

The microbiology team used this report as evidence and took it to the medical superintendent of the hospital to drive policy actions, such as requiring trained medical interns to be primarily responsible for sample collection and ensuring all sample details were filled in the requisition form before they were accepted for testing. The microbiology team also changed the format of the requisition form where they highlighted the mandatory information which was required to be filled before the sample was accepted. The software team then automated the process of report generation and now a monthly report is generated and reviewed monthly to understand what gaps persist. A data quality report generated two months after the workarounds were implemented at the hospital is shown in **Table 2**, reflecting a significant improvement in completeness, with now less than 20% of the fields being incomplete as compared to 70-80% previously.

**Table 2:** Comparison of data quality over a year.

Period	Out patient count	Microbiology investigations	Tests/Out patient count (%)	Total investigations	Tests/total tests (%)
Yearly average	641212	14462	2.26	718097	2.01
Monthly average	45291	1257	2.78	67219	1.87
Daily average	2313	52	2.25	2868	1.81
Comparison to the previous year					
May 2021	15923	927	5.82	35685	2.60
May 2022	49324	1826	3.70	78548	2.32

### 3.3 Practices to enhance the circulation of this visibility across the health institution

**Figure 2:**
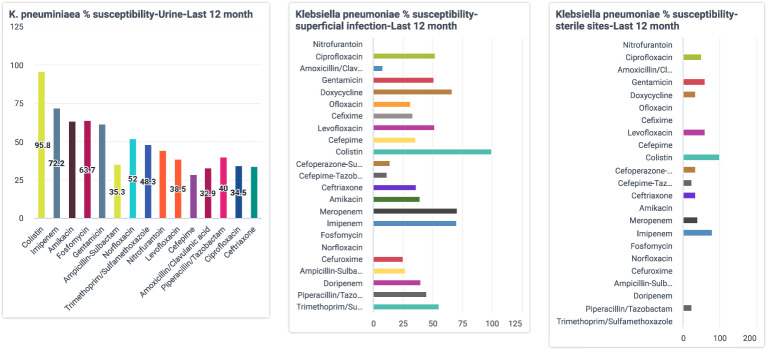
A sample dashboard.

As the data entry processes were increasingly streamlined, the attention shifted to the outputs and the microbiologists started giving requirements for their desired data outputs. These requirements were discussed with the software team, developed, and made available through the customized dashboards offered by the digital platform. The microbiologists could now independently download the automated reports from the application dashboard, which was updated daily with current data, and a stack of reports started to be compiled and emailed to the hospital management, such as the Antibiotics Stewardship Committee, the clinical departments, and also the State Secretariat. Some of the reports on the dashboard are shown in **Figure 2**, including an aggregation of the sample types received at the lab, organisms identified from the samples, and the antibiotics sensitivity pattern.

A microbiologist mentioned the utility of these reports in day-to-day work:

*It used to take us weeks to count the numbers from the register and prepare a report. Sometimes, we even skipped developing this report because it was cumbersome and practically impossible to count several figures and document them correctly.*

**Figure 3:**
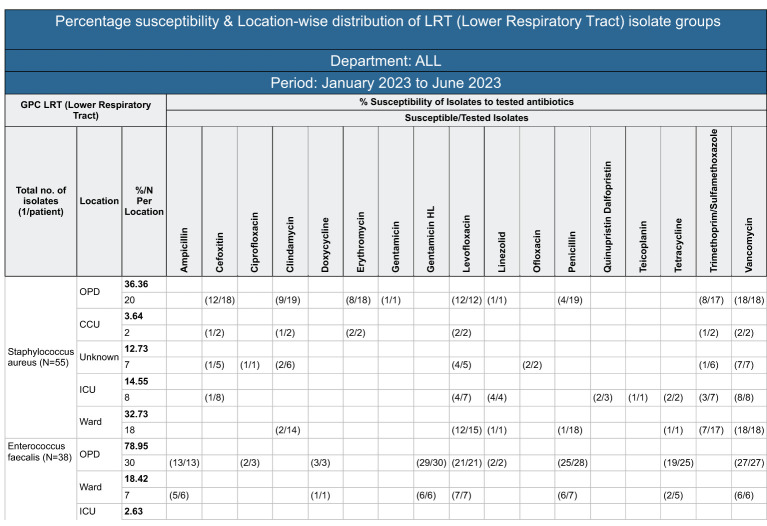
A sample department-wise report.

But now with support from the digital application, the microbiology lab saw the potential of evolving the existing quarterly susceptibility report into a quarterly organization-wide susceptibility report. After many rounds of discussions, it was decided to develop an aggregate report with department-wise trends of resistance, which could potentially help physicians to decide on the antibiotics to be prescribed based on the sensitivity patterns highlighted by the test reports. The microbiology lab took 4-10 days from the day of sample collection to the generation of the test report. This was too long, and in the meantime, the physician would end up prescribing broad-spectrum, higher-generation antibiotics to provide immediate care to the ailing patients. With this report, the team identified that physicians could make better evidence-based decisions, even in the absence of immediate test results. A sample monthly resistance report sent to all physicians is shown in **Figure 3**.

Another new practice introduced was in the process of displaying the test results. The existing manual registers did not have the fields (and space) to capture details of the department, location by month and year, and to also fit in one page the long computer registration number. To resolve this issue, the microbiology team decided to design stamps with a predefined list of antibiotics for each sample and organism to be printed in the register to minimize manual data entry by the lab technicians, maintain data quality by standardizing the output, and in saving time to circulate the results. As a future practice modification, the microbiology team has also decided to change the format and reprint the registers to capture the necessary information after finishing their existing printed stock of registers. For example, the existing register does not have the fields for departments (Medicine, Surgery, etc.) and location (Outpatient department, Inpatient department, Intensive care unit etc.) The format of new registers is being defined with the data set to be analyzed in consideration.

### 3.4 Practices to act on this information for changing policy and practice, within and across hospitals

As the microbiology team started generating their reports in their desired formats, the focus shifted to enabling the use of the data to build greater awareness about the problem of AMR within the hospital. As data entry processes improved, the microbiology team identified clinically relevant data to be reported to the physicians and the hospital management. For example, the microbiology team included age and gender-specific data to identify the age and gender-specific resistance. Another reporting format was developed which identified the top three resistant and sensitive isolates in the hospital.

#### 3.4.1 Dissemination of information to the treating physicians

The monthly and quarterly department-wise reports sent to all treating physicians at the hospitals can potentially guide them to prescribe antibiotics therapy. Interviews were conducted with the physicians at various departments in the outpatient departments, wards, and ICUs to understand how these reports can be improved to better meet their needs. A consultant from the Gastroenterology department said:

*I always wondered if I had such a report available while prescribing, I would have an idea of what antibiotics are susceptible and resistant in the area. Currently, we prescribe a second or third line of antibiotics during the first consultation, but it is better to prescribe the first-generation antibiotic if it is susceptible in almost 80% of cases as I see in this report. However, I did not think anyone would take an initiative to make such data available at a public hospital.*

#### 3.4.2 Dissemination to the antimicrobial stewardship (AMS) committee and state

**Figure 4:**
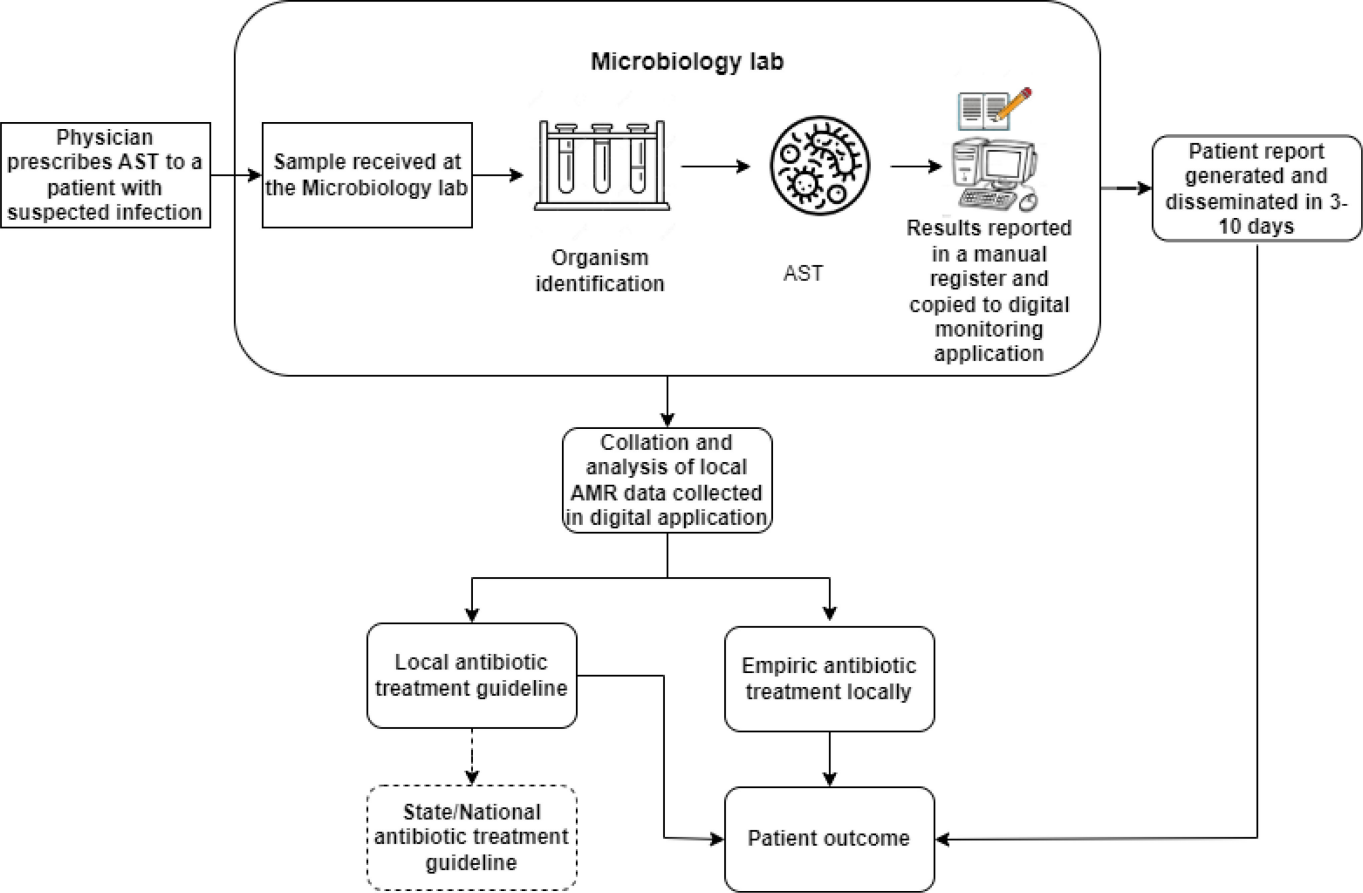
Information flow after the interventions and data use efforts.

As per the national guidelines, all public hospitals were mandated to form an antimicrobial stewardship committee. The hospital formed a committee in October 2021, which is led by the Head of microbiology and includes members from other departments, such as Medicine and Surgery. The committee meets every month, and the HOD microbiology presents the monthly resistance profiles downloaded from the monitoring application to the members. The committee decided to use one year of resistance profile report data to develop an infection control and antibiotics use policy for the hospital. Additionally, the hospital shares a monthly resistance profile with the AMR coordinating centre in the state. A depiction of the flow of information with these interventions is shown in **Figure 4**.

This process is now ongoing with the research team to have discussions with the different teams, understanding their information needs identified in reference to the current organization-wide report format, and for the technical team to provide the required data support.

In an annual workshop organized in July 2022, the new hospital that had adopted the application updated the progress of data entry and AMR data management. This time the physicians from all clinical departments also attended the workshop to discuss the possibility of using the resistance data effectively, particularly for patient care and policy making. All departments in the meeting after the workshop were directed to develop a department-wise guideline based on the reports they received and submit it to the department of Pharmacology. The microbiologists showed their reports from the last three years to all department heads attending the meeting. The chief pharmacist and team were made responsible for developing and presenting a hospital antibiotic policy in consideration of the guidelines shared by all departments. The hospital antimicrobial stewardship committee was thus tasked to develop a local antibiotics policy with guidance from experts from other hospitals who have developed this policy. This stage had its challenges, and further work is required to create the policy and implement it.

Additionally, a baseline AMR application with minimum data set for AMR monitoring was maintained by the technical team with basic features to report, monitor, and analyse AMR data. This allowed the implementation of the application in other contexts with minimal development requirements. At the same time, new requirements, like the Android reporting and new reports developed at other sites, have been incorporated for new facilities, and some of them have been made available to the original facility to add greater value to their processes. Having data reported on standard parameters also helped in extending the learnings from this hospital to other medical college hospitals in Himachal Pradesh state to strengthen the state-wide reporting system.

## 4 Discussion

The process of developing an evidence base for action in LMICs is a multi-stage process that is conceptualized through three interconnected phases: input, output, and outcome. This is conceptualised as a process perspective for developing a digitally mediated data driven policy.

### 4.1 Digitally mediated data driven AMS at local level

The first phase, input, involves the stabilization of data entry, ensuring that the data collected is reliable and accurate. A regular monitoring of the data entry and validation of the data collected in the manual registers and the digital application is essential. The second phase, output, involves the generation of outcomes, where data is analysed and transformed into useful information for decision-making. Finally, the third phase, outcome, involves the use of information to initiate conversations around data and take action at the local level. This process began with digitising and stabilising the data entry practices on the input side and then constructing outputs. These outputs were the foundation for developing outcomes using data to guide policy and practice decisions. This process necessitated continual, interdependent, and iterative cycles that built upon the inputs, fed into the outputs, and ultimately led to the articulation of outcomes. This process is conceptualised analytically as three interconnected stages, and the process, leading to organisational outcomes of a digitally enabled institution for AMR data management relevant to an LMIC context.

The importance of digital surveillance in promoting responsible use of antibiotics cannot be overstated. This paper emphasizes the crucial role of digital technology in healthcare facilities with resource constraints to promote responsible use of antibiotics. This information can be used to develop preventative strategies to improve antibiotic stewardship and reduce the emergence of antibiotic-resistant bacteria. However, the process of designing, implementing, and routinizing digital surveillance technologies in resource-constrained settings can be challenging. It goes through a process of building local capacities to initiate conversations around data and building skills to manage and analyse data effectively. It also involves raising awareness about the use of digital technologies and preventative strategies among healthcare professionals and stakeholders. To overcome these challenges, the paper argues for building local capacities to develop effective and sustainable antimicrobial stewardship (AMS) programs. This involves i) enabling networking around digital solutions, ii) creating value in networked partnerships, iii) initiating conversations around data, and iv) raising awareness of the digital to develop AMS programs.

### 4.2 Enabling networking around digital solutions and data

Drawing from the understanding of context-specific challenges, by understanding the processes and mechanisms for building a network of collaboration among multiple stakeholders through digital interventions. This research describes how these networks are slowly and incrementally built and evolved through sharing knowledge and collaborative work. The collaboration between HISP India and the hospital team started as a system development exercise with the microbiologists, which slowly evolved as they participated in the design of the antibiotics stewardship management policy involving the clinicians and hospital administration.

Building capabilities of the hospital staff around the digital tools was key in enabling intra-hospital and inter-hospital networking. As they became more confident of the digitally analysed testing data, the microbiologists could speak up strongly in the antibiotics stewardship committee meeting and build the basis for the principal to declare what he wants for the hospital: “a data-driven locally relevant antibiotics stewardship policy”. In workshops, which microbiologists from the neighbouring hospitals also attended, the reference hospital could proudly demonstrate their work and encourage the other hospitals to initiate similar processes in their settings. The underlying message conveyed in these interactions was “if we can do it, even you can do it.”. Today, one other hospital has established and is routinely running a similar digital system, and two others are ready to start once their lab infrastructure is established. The timeframe to operationalise this scaling was much shorter than in the first hospital, as they could benefit from the earlier learning.

### 4.3 Capacity building through mutual learning

The digital intervention goes through cycles of refinements ranging from major to minor design changes after initial implementation based on identifying organisation-specific requirements. This ongoing design and implementation followed ‘learning by doing’, which practically contributed to building the hospital team’s technical and adaptive capacity through mutual learning and knowledge exchange and translation. The mutual learning approach recognises that knowledge has to be always shared both ways: HISP team learnt about the domain of AMR and data handling practices of the hospital; the hospital staff needed to learn about the functioning of the digital tool, what it can and cannot do, and how best it can add value to their work.

In the initial design, the microbiology team could not coherently describe their requirements, but as they saw the system and understood its potential, they could slowly start telling their needs, which increasingly became more sophisticated over time. Similarly, as the NGO learned more about the hospital work, they could also make suggestions for features to be incorporated into the application. For example, as the microbiologists described their analysis needs, the NGO pointed out the current registers don’t record the particular data (for example, the patient’s location). The microbiologists then took the proactive step of writing to the principal, requesting permission to reprint the registers incorporating the necessary data fields. Highlighting mutual learning, one microbiologist mentioned, *‘The development of the digital system is a process of learning for us as well. We are not only learning about digital management of AST test results and how to use the system but also identifying new ways to improve our processes as we face a challenge with the system,’*

### 4.4 Initiating conversations around the data

The research on health IT in LMIC contexts has forever lamented the problem of “too much data and too little use” (Sahay et al., 2017). Often, a solution to this problem is to build more shiny dashboards and digital tools, and current debates are extolling the virtues of big data and machine learning (Schwalbe and Wahl, 2020). This represents a response based on rational principles that more data will lead to more (and better) action, an assumption that has been discounted by research (Sahay et al., 2017). This paper describes that the focus on digital enhancements on their own, which tend to focus on data as an end product or commodity, is inadequate. It is rather important to understand the processes by which data is collected, quality-assured, analysed, and circulated. Each stage of this process involves different conversations around data concerning different actors. In this research, at the data entry level, it was between the data entry operator, the microbiology lab staff, and HISP India, while at the stewardship management committee, it was between the principal, the clinicians, and microbiologists.

This paper highlights how these conversations around data provide the basis for action. For example, when a gynaecologist was presented with an organisation-wide report showing the resistance pattern, she realised that in the future, she would consider prescribing a first-generation antibiotic to patients with Urinary Tract Infections instead of the previous practice of prescribing a second-generation antibiotic. For the NGO team, regular conversations with the hospital staff provided them with the basis to improve the design and continuously provide the hospital with upgraded functionalities. Identifying actions to be taken often raises the need for new conversations, such as of the principal with the clinical departments, on how the policy can be made more relevant to them. Employing WHO AWARE (Access, Watch, Reserve) scale is an important tool to streamline the use of antibiotics and for the development of antimicrobial stewardship policy. WHO AWARE scale highlights the importance of matching prescriptions to the infectious agent. By doing so, healthcare professionals can ensure targeted treatment, preserve the effectiveness of antibiotics, and promote patient understanding and adherence. These practices are fundamental in combating AMR and preserving the efficacy of antibiotics for future generations. 

### 4.5 Raising awareness of the digital in its mitigation

Antibiotics use, and prescriptions represent complex bio-social processes driven by several medical, social, cultural, and economic conditions. In LMICs, with existing challenges of capacity and resources, creating awareness about “new” problems when hospital staff is already preoccupied and overburdened with existing challenges is a complex undertaking. While the global and national levels have developed various guidelines and policies around AMR, these tend to be poorly understood and translated into hospital settings of LMIC contexts (Charani et al., 2023). This translation requires deeply contextualised and intensive institutional work, including building awareness of the problem and potential solutions to address them. The microbiologists were identified as key actors in this process of making AMR data visible and working closely with them, relevant digital solutions were designed and made to work to support different processes of data entry, data quality, analysis, and dissemination of analysed reports. These processes helped raise awareness of the problem incrementally, first among the microbiologists and then others in the hospital. Further, it was demonstrated that it is not all hopeless, as digital technologies supported by collective and collaborative actions can contribute to finding some local solutions.

Through this analysis this paper makes two key contributions toward engaging with the practical challenge of raising awareness. One, top-down guidelines and directives tend not to work at the micro-level since actors are preoccupied with their local everyday challenges. These directives need to be translated and made relevant to these actors in the context of their everyday work. Two, since there are multiple constituencies involved, a common thread for building awareness needs to be identified and nurtured. This common thread, in this case, related to digitally generated AMR testing data, which was required by the microbiologists, the clinicians, the hospital administration, and the patients. The digital solution thus served as an important vehicle to build awareness and mobilise the intentions of collective action. This initiated a process of building a shared understanding of the “why” of the institutional work.

### 4.6 Creating value in networked partnerships

This research helped to identify that the value proposition for different actors within a heterogenous group varies, and it needs to be approached incrementally and with different means. For the microbiologists, it gave them more control over the testing data by reducing the burden of their everyday manual work and allowing them to focus on data analysis, which was a source of professional pride. Further value came to them as they found more confidence and voice to express their findings in the hospital-wide stewardship management meetings. The principal of the hospital, who was responsible for implementing the state mandate of creating effective stewardship management, saw value in how the organization-wise infection analysis could help in building a data-driven policy. Achieving this would no doubt help showcase his hospital in the rest of the state. However, it must be emphasised that building this policy is not a given but will require further and intensive collaborative institutional work. Value for the clinicians came as they could use the analysis reports to do better clinical therapy. This, too, is not a given as there are disagreements between disciplines, such as between paediatricians and surgeons, about the value of data for guiding clinical therapy. These differences must also be negotiated over time, and data must be better customised for different needs. For the NGO, the value came with building unique digital innovations in areas that were to date unchartered. Their experiences in this hospital opened the doors for projects in other states and countries. This analysis resonates with the comment by (Selznick, 2011) that institutions are *“infused with value, that is prized not as tools alone but as sources of direct personal gratification and vehicles of group integrity”.*

The associated stakeholders need to derive value from work, which needs to be developed incrementally and over long periods. The value developed for the microbiologists further yielded value for other constituencies within the hospital (such as the principal and physicians), who then initiated workshops that other hospitals could attend and adopt the digital systems. With time, the aim is to develop a state-wide reporting network, making it pioneering in the country. Also, this paper describes that this value is not a given and will never be static, but would need to be constantly created, maintained, and disrupted. For example, the HISP India needs to continually upgrade and innovate digital solutions to meet the diversity of emerging information needs. The principal needs to constantly negotiate with the different actors in his hospital, for example, the paediatricians and surgeons, to work within the framework of a data-driven policy. Such cumulative value development will necessarily take place over extended periods.

### 4.7 Regular operational research

Regular operational research activities play a crucial role in monitoring the progress of Antimicrobial Resistance (AMR) monitoring efforts. These activities provide valuable insights and data that help assess the effectiveness of interventions, policies, and strategies aimed at combating AMR. By employing rigorous research methodologies, such as data collection, analysis, and evaluation, operational research activities enable the tracking of key indicators related to AMR, including the prevalence and patterns of antimicrobial use, resistance rates, and the impact of interventions on reducing AMR. This information allows policymakers, healthcare professionals, and researchers to make evidence-based decisions, identify areas of concern, and develop targeted interventions to address the challenges posed by AMR effectively. Furthermore, regular operational research activities help identify emerging trends, evaluate the implementation of control measures, and contribute to the global knowledge base on AMR, thereby facilitating continuous improvement and informed decision-making to mitigate the growing threat of antimicrobial resistance

## 5 Conclusion

This paper highlights the multi-stage process of developing an evidence base for action in LMICs, using the example of developing a digitally mediated data-driven policy for AMS. The three interconnected phases of input, output, and outcome, are crucial in ensuring the reliability and accuracy of data collected, generating useful information for decision-making, and taking action at the local level. The paper emphasizes the importance of digital surveillance in promoting responsible use of antibiotics and building local capacities to develop effective and sustainable antimicrobial stewardship programs. Enabling networking around digital solutions and data, building capabilities of hospital staff around digital tools, and initiating conversations around data are crucial in capacity building and mutual learning. Finally, the paper argues that digital interventions must go through cycles of refinements based on identifying organisation-specific requirements and promote mutual learning to ensure effective implementation and sustainable impact.

## Data availability statement

The raw data supporting the conclusions of this article will be made available by the authors, without undue reservation.

## Author contributions

The author designed the study, performed the research including and data analysis, and wrote the manuscript.

## Footnotes

1 Antimicrobials are a broad category of drugs used to treat infections caused by microorganisms such as bacteria, viruses, fungi, and parasites. They include antibiotics, antivirals, antifungals, and antiparasitic drugs.

2 The National Centre for Disease Control (NCDC)- 35 laboratories in state medical colleges, Indian Council of Medical Research (ICMR)- 30 tertiary laboratories and WHO Country Office for India with the Indian Association of Medical Microbiologists Delhi (IAMM-DC) to establish the WHO-IAMM Network for Surveillance of AMR.
